# Designing Aptamer-Gold Nanoparticle-Loaded pH-Sensitive Liposomes Encapsulate Morin for Treating Cancer

**DOI:** 10.1186/s11671-020-03297-x

**Published:** 2020-03-30

**Authors:** Xiaoyuan Ding, Chenyang Yin, Weiwei Zhang, Yu Sun, Zhenzhen Zhang, Endong Yang, Dongdong Sun, Weiyun Wang

**Affiliations:** 1 School of Life Sciences, Anhui Agricultural University, Hefei, 230036 China; 2 School of Biochemical Engineering, Anhui Polytechnic University, 8 Zheshan Road, Wuhu, 241000 Anhui China

**Keywords:** Morin, Aptamer, Gold nanoparticle, pH-sensitive liposome, Anticancer

## Abstract

This study proposes the synthesis of a type of anticancer nanoparticle, aptamers and Au nanoparticle (Apt-Au)-modified Morin pH-sensitive liposome (MSL), which exhibits targeting properties. Tumors are difficult to cure because their microenvironment varies from that of normal tissue; its pH is lower than that of normal tissue, which generally impedes the effectiveness of drugs. Thus, pH-responsive drugs have attracted extensive attention. Gold nanoparticles (AuNPs) show potential as drug carriers because of their small size, good biocompatibility, easy surface modification, and strong cell penetration. Apt-Au@MSL exhibits excellent monodispersity and tumor-targeting properties and can be released in partly acidic environment via dialysis. We screened our model cancer cell by MTT assay and found that SGC-7901 cells can effectively suppress proliferation. In vivo results demonstrate that the administration of Apt-Au@MSL could inhibit tumor growth in xenograft mouse models. H&E staining and TUNEL assay further confirmed that Apt-Au@MSL can promote tumor apoptosis. Apt-Au@MSL may induce apoptosis by triggering overproduction of reactive oxygen species (ROS) and regulating multiple signal crosstalk. Both blood biochemistry tests and H&E staining suggested that these materials exhibit negligible acute toxicity and good biocompatibility in vivo. With its powerful function, Apt-Au@MSL can be used as a target-based anticancer material for future clinical cancer treatment.

## Introduction

One of the most prevalent causes of death [1], cancer, can lead to substantial economic loss and harm to humans. Considerable efforts have been directed toward the development of smart drugs for cancer treatment [2]. Owing to their capability to protect drugs until they reach the target site, polymer nanoparticles can potentially improve the delivery of therapeutic drugs [3]. To ensure that the therapeutic agent is delivered to the active site, highly reactive nanoparticles are used. Such nanoparticles can be designed to vary in material properties under different biological stimuli. Reactive nanoparticles are designed using different stimuli, including external stimuli (e.g., temperature and light) and biological stimuli (e.g., pH or redox conditions). NPs responsive to pH have attracted research interest because of changes in pH after nanoparticle endocytosis. Materials that respond to pH also draw attention because the pH-responsive function can be easily integrated into a range of polymer structures to design a set of nanoparticles that respond to pH. Nanoparticles can respond to pH by alteration of surface chemistry, change in particle size or shape, and breakdown or release of substances. This change in nanoparticle properties can be used to regulate cellular uptake and controlled release. Therefore, pH-responsive nanoparticles provide a powerful strategy for the design of therapeutic delivery systems.

**Fig. 1:**
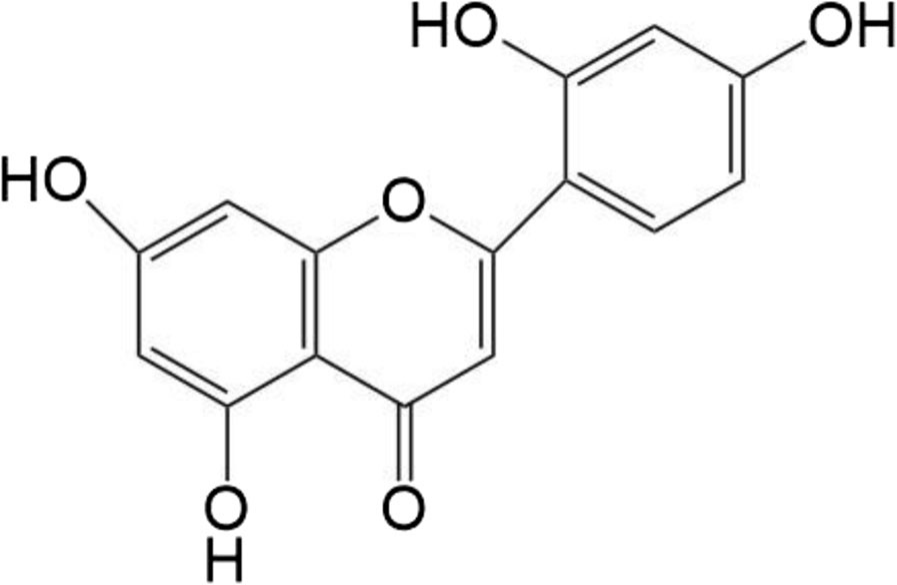
Chemical structure of Morin hydrate

Morin hydrate (3,5,7,2′,4′-pentahydroxyflavone) (Fig. 1) is a natural active substance isolated from Chinese herbal medicines or plants [4]. It is a progesterone compound and a secondary metabolite of phenol in plants. Morin is widely distributed in nature and exerts good antioxidant, anticancer [5], and significant anti-inflammatory effects. The potential of Morin for various applications has drawn considerable interest [6], but such potential is significantly limited by the low water solubility and bioavailability of the substance [7].

Liposomes (hollow) composed of lecithin and ceramide, among others, have a bilayer structure consisting of amphiphilic lipid molecules [8]. Liposomes provide desirable features, such as biocompatibility, functionality, reduced side effects, and ability to encapsulate large amounts of medicine [9, 10]. They can efficiently carry hydrophilic and hydrophobic agents and protect them from external conditions, successfully transporting them to targeted tissue regions [11]. Improved efficacy is incorporated in the design to facilitate the pH-triggered release of the anticancer material within the tumor interstitium via pH-sensitive liposomes [12–15]. These pH-sensitive liposomes are special liposomes that release drug in tissue environments and rapidly destabilize in acidic environments, such as endosomes of cancer tissue [16–18].

For enhanced selectivity and efficacy of tumor treatment, pH-sensitive liposomes should be modified to form tumor-targeted nanoparticles [19]. DNA aptamers have recently been developed as highly selective and sensitive biosensing and imaging sensors, as well as potential agents for cancer-targeted therapeutics [20]. The single-stranded DNA aptamer AS1411 has been shown to function as a chemotherapeutic agent because of its high binding affinity for cancer cell nuclei [21]. This aptamer (Apt) was combined with Au NPs to fabricate a two-component nanoconstruct of Apt-loaded Au NPs that can interact with cancer cell nuclei. Some studies have also demonstrated that the design of liposome-nanoparticle hybrids can provide a rich toolbox for the fabrication of such multifunctional modalities [22]. A hybrid liposome–Au NP vesicular system consisting of cationic liposomes and Au NPs has been designed to improve drug penetration within the tumor interstitium and enhance anticancer activity [23, 24].

Gold nanoparticles (Au NPs) have been regarded as good drug carriers and can be modified with bio-related molecules to enhance cancer-targeted specificity [25]. With the surface modification of Au NPs, the aptamer of the sulfhydryl group can be used to modify the surface of Au NPs, which can be targeted by the Au-S covalent bond, and the appropriate sulfhydryl group on the nanogold surface can be attached to the nanogold surface [26]. From an engineering and application perspective, ligand-bound gold nanomaterials provide a powerful platform to facilitate targeted identification, detection, and treatment.

**Fig. 2:**
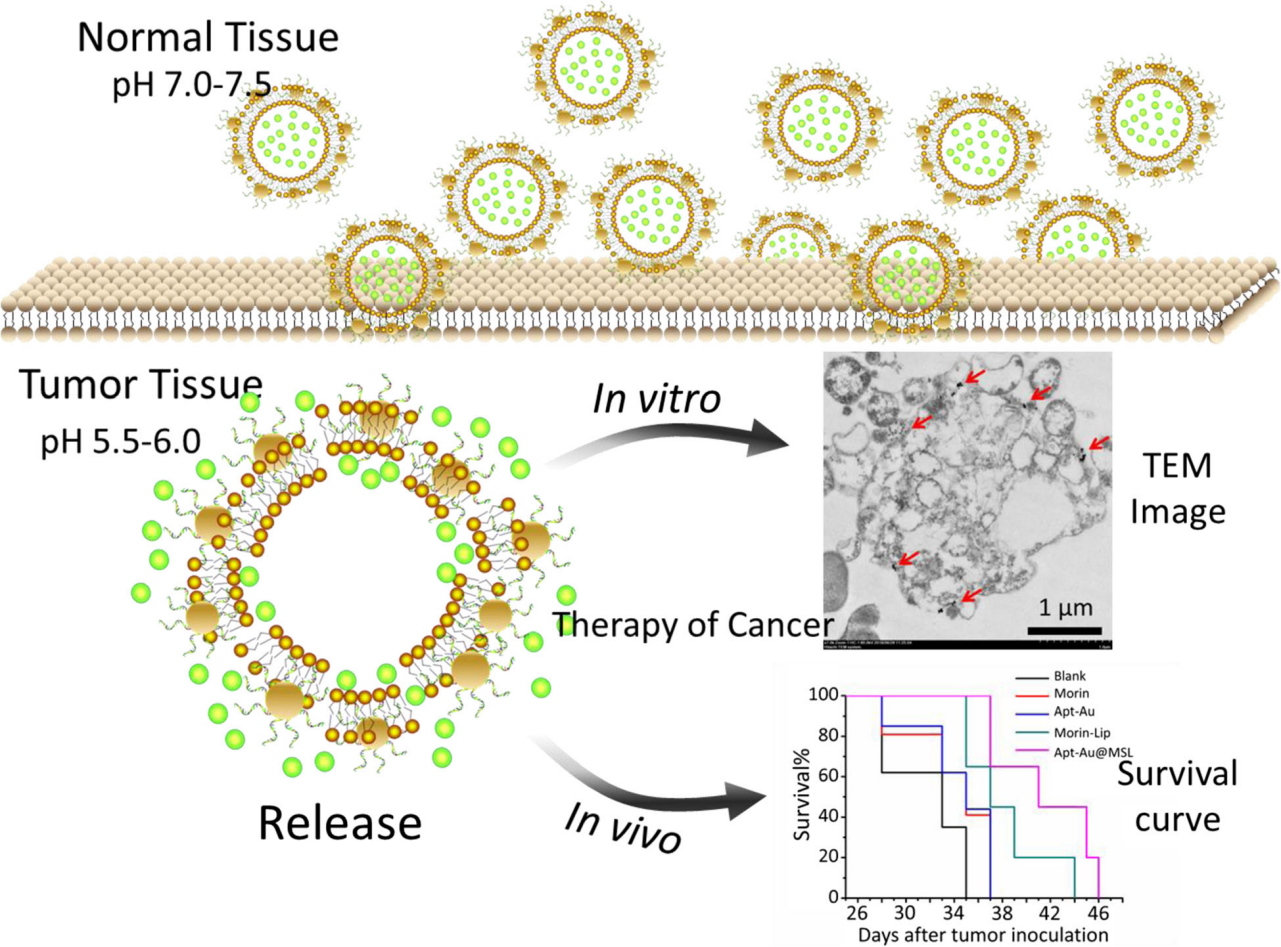
Proposed schematic diagram of our designed Apt-Au@MSL containing Morin for drug delivery to cancer cells with tumor-targeted property.

**Fig. 3:**
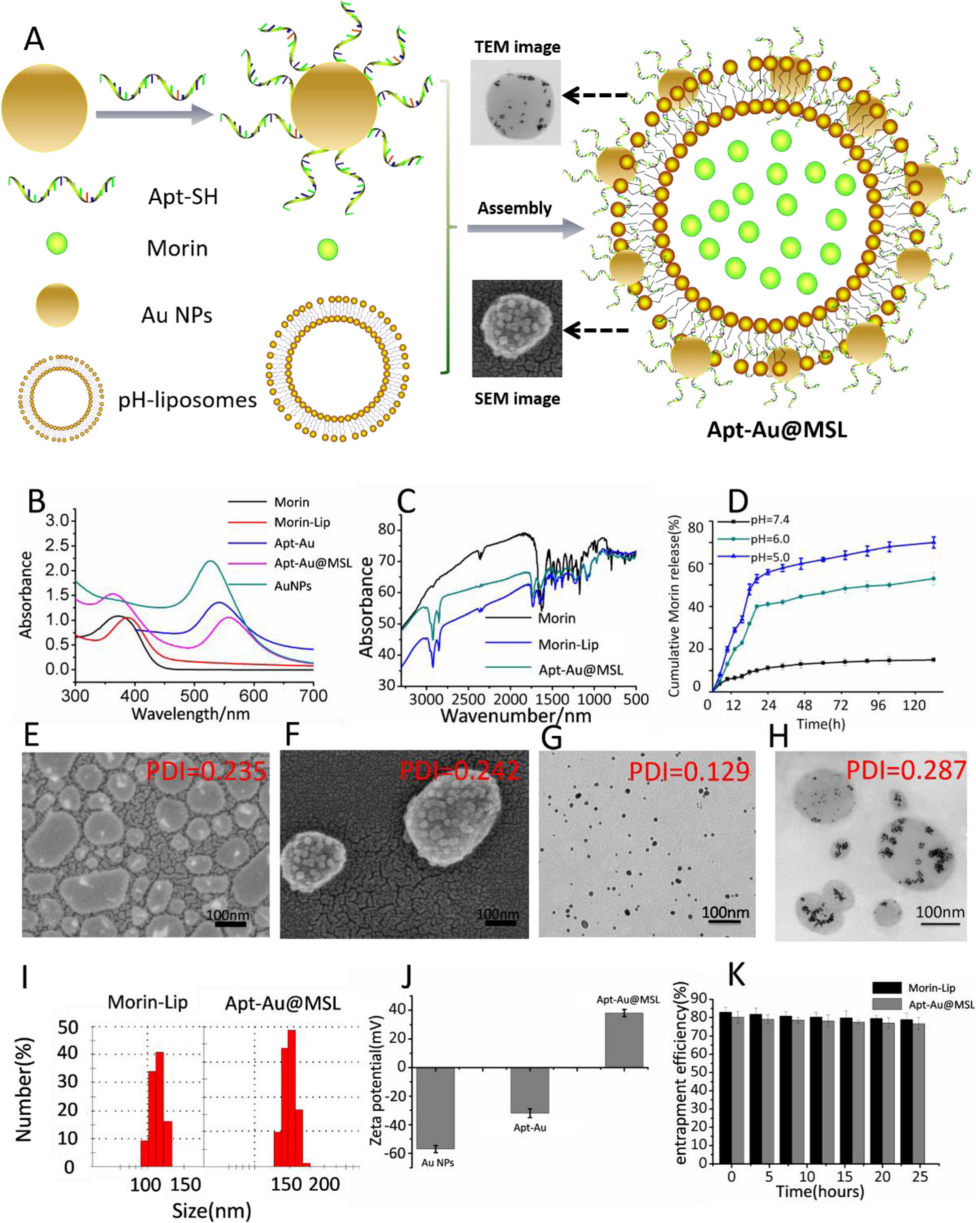
Characterization images. **a** Schematic illustration of the synthesis of Apt-Au@MSL. **b** Ultraviolet absorption spectra of Morin, Au NPs, Au-Apt, Morin–liposome, and Apt-Au@MSL. **c** FT-IR spectrometers of Morin, Morin–liposome, and Apt-Au@MSL. **d** The release behavior of Morin from Apt-Au@MSL at different pH conditions. **e** SEM image of the Morin–liposome. **f** SEM image of the Apt-Au@MSL. **g** TEM image of the Au NPs. **h** TEM image of the Apt-Au@MSL. **i** Diameters of Morin–liposome and Apt-Au@MSL determined at least thrice via DLS. **j** ζ-Potential of Au NPs, Apt-Au, and Apt-Au@MSL. **k** Entrapment efficiency rate (EE%) of Morin–liposome and Apt-Au@MSL

In the current study, we used pH-sensitive liposomes encapsulating Morin and assembled Au-Apt that was modified on the surface of the liposomes (Apt-Au@MSL, Fig. 3a). Morin, which is characterized by low water solubility and bioavailability, was transformed. The morphology, size, and other properties of the prepared Apt-Au@MSL were characterized. The new nanoparticle showed tumor-targeted properties and high anticancer activity. The anticancer activity of Apt-Au@MSL was explored in vitro and in vivo (Fig. 2).

## Materials and Methods

### Materials

L-α-Phosphatidylcholine (PC), cholesterol (Chol), cholesteryl hemisuccinate (CHEMS), Morin (≥ 99.99%), sodium citrate (99.9%), HAuCl_4_·3H_2_O (≥ 99.9%), polyvinylpyrrolidone (PVP, wt 40,000), NaOH (≥ 98.0%), and HCl (37%) were purchased from Sigma-Aldrich Chemical Co. (USA). The AS1411 aptamer with a disulfide modification was synthesized by TaKaRa (Dalian, China) with the following sequence: 5′-HS-T-(C6-S-S-C6)-TTG GTG GTG GTG GTT GTG GTG GTG GTG G-3′. Thiazolyl blue tetrazolium bromide (MTT), propidium iodide (PI), calcein, and Alexa Fluor 488 Annexin V were purchased from Sigma-Aldrich Chemical. All aqueous solutions were prepared in doubly distilled water. All other reagents were the best commercially available.

### Synthesis of Au NPs

Up to 1 mL of HAuCl_4_·4H_2_O (1%) was dissolved in 25 mL of Milli-Q water (pH = 5.5), and 50 mg of PVP was added into the solution. The obtained solution was poured into a three-necked flask (150 mL) and was treated by microwave (MW) irradiation for 5 min with mechanical stirring. Subsequently, 1.5 mL of sodium citrate solution (1%) was quickly added into the solution and was MW-irradiated for 3 min. The solution was collected by centrifugation at 10000 rpm for 5 min.

### Synthesis of Apt-Au NPs

The disulfide bond of the aptamers was cleaved using tris(2-carboxyethyl)phosphine. After 30 min, the aptamer (100 μL, 100 μM) solution was added to 10 mL 5 nM solution of Au NPs and then incubated for 24 h to form Au-Apt NPs. After 1 day, we salted the mixture solution with 2.5 mL of a 500 mM solution of NaCl twice, separated by 4 h [27].

### Preparation of pH-Sensitive Liposomes

Liposomes composed of egg PC to CHEMS to Chol at the molar ratio of 33:13:5 and 5% Morin were prepared by film hydration. Egg PC, CHEMS, Chol, and 2 mg Morin were dissolved in 8 mL CHCl_3_, and the organic solvents were removed at 40 °C with a rotary evaporator. For Morin–liposomes, the resulting thin lipid film was hydrated using 2% (w/v) PBS and then sonicated using an ice bath probe for 15 min. The final liposome was synthesized and stabilized in the colloidal system. For Apt-Au@Morin pH-sensitive liposomes (Apt-Au@MSL), the lipid film was hydrated with 475 μg/mL Au-Apt NPs in 5% dextrose followed by sonication [23].

### Characterization

Ultraviolet–visible spectroscopy (UV–Vis, S-3100 Photodiode Array, Scinco Co., Ltd., Korea) and Fourier-transform infrared spectroscopy (FT–IR) were conducted (Nicolet iS50, USA, Thermo Fisher Scientific). The morphology of the Apt-Au@MSL was examined by transmission electron microscopy (TEM, HT7700, Tokyo Japan, Hitachi). The release percentage of Morin was detected by UV–vis spectroscopy. The morphology of the Morin–liposomes and Apt-Au@MSL were also observed by scanning electron microscopy (SEM, S-4800, Hitachi, Japan). Dynamic light scattering (DLS) and zeta potential measurements were used to characterize the optical properties and sizes of Morin–liposomes and Apt-Au@MSL on a Brookhaven ZetaPALS potential analyzer.

### Cell Culture

The human cancer cell lines, SGC-7901, BGC-823, A549, HeLa, MCF-7, and Hs68, were purchased from American Type Culture Collection (ATCC) and maintained in RPMI with 10% fetal bovine serum at 37 °C and 5% CO_2_.

### In Vitro Anticancer Activity Study

SGC-7901, BGC-823, A549, HeLa, MCF-7, and Hs68 were seeded on 96-well plates (2.0 × 10^3^ cells/well) and then cultured with Apt-Au@MSL at varying concentrations (0, 5, 10, 15, 20, and 30 μg/mL). Au NPs and Morin were assigned as the control groups. The blank group consisted of untreated cells. The 96-well plates were incubated in a humidified incubator. After incubation, 9.6 mL of MTT solution (5 mg/mL) was added into each well and further incubated for 2 h. Each group was tested in triplicate, and IC_50_ values were derived from the mean OD values of the triplicate tests versus drug concentration curves. The brightness of each group was obtained by confocal fluorescence microscopy [28].

Cell adhesion was monitored using a real-time cell electronic sensing system (RT-CES; ACEA Biosciences, Inc.) every 10 min for 75 h. To determine cell adhesion, each well in the plate was seeded with cells (1.0 × 10^4^ cells/well) with a fresh medium to a final volume of 200 μL and then incubated in an Apt-Au@MSL solution at varying concentrations (10, 20, and 30 μg/mL) at 10 h incubated for 12 h [29]. The blank group consisted of untreated cells, and the control groups consisted of the Morin and Morin–liposome groups.

### Fluorescence Microscopy

SGC-7901 cells were grown in the 48-well plates overnight at 37 °C, 5% CO_2_. The cells were then treated with Apt-Au@MSL solutions (10 and 30 μg/mL) at different concentrations for 12 h. The control groups consisted of the Morin and Morin–liposome groups. Subsequently, the medium was removed. The blank group consisted of untreated cells. The cells were washed with PBS three times. The cells were stained and then measured by co-staining of live and dead cells (the LIVE/DEAD assay). After co-staining with Calcein–AM/PI for 30 min, the cells were washed with PBS twice to remove the excess dye, and fluorescence images were obtained by confocal laser scanning microscopy (CLSM) (for calcein–AM, Ex = 488 nm and Em = 515 nm; PI Ex = 535 nm and Em = 615 nm [30]).

### Apoptotic Cell Death Analysis

To measure the anticancer effects of Apt-Au@MSL, each well in a six-well plate was seeded with SGC-7901 cells (1.0 × 10^5^ cells/well) and then incubated in Apt-Au@MSL solutions at varying concentrations (5, 10, 20, and 30 μg/mL) for 12 h. The blank group consisted of untreated cells, and the control groups consisted of the Morin and Morin–liposomes groups. After treatment, the cells were collected and washed with PBS twice. The cells were stained with PI and Annexin V–FITC and then analyzed using a CytoFLEX analyzer (Beckman Coulter) (for PI, Ex = 535 nm and Em = 615 nm; Annexin V–FITC, Ex = 488 nm and Em = 525 nm) [31].

### Studies on Wall Destruction

To study cell wall destruction in anticancer assay, the SGC-7901 cells were grown in six-well plates at 37 °C and 5% CO_2_. The cells cultured without the material were used as the blank group. After the cells were cultured with Apt-Au@MSL at varying concentrations (5, 10, 20, and 30 μg/mL) for 12 h, they were treated with Trypsin–EDTA solution 0.25%. All cells (including the floating cells in the medium) were collected with 2000 r/min for 5 min. The collected cells were then post-fixed in 2.5% glutaric dialdehyde solution for 4 h [32]. The fixed cells were dehydrated in an acetone gradient series for 20 min. The cells were ultimately subjected to a series of procedures, mounted on copper grids, and observed by TEM (HT-7700, Hitachi, Japan).

### Western Blot Assay

The protein expression of SGC-7901 cells was detected by Western blot analysis. SGC-7901 cells (4.0 × 105 cells/well) were inoculated on a 9-cm culture plate and 20 μg/mL Apt-Au@MSL for 1, 6, 12, and 24 h. Cells were harvested after treatment and suspended in a cell lysis buffer on ice for 1 h. The mixture was placed in a centrifuge at 11,000*g* at 4 °C for 10 min, and the supernatant containing total cellular protein was collected. Total protein concentration in the supernatant was determined using the BCA method. The protein was then resuspended in the loading buffer and boiled at 100°C for 10 minutes. Samples containing equal amounts of protein (40 μg/lane) were subjected to SDS-PAGE (10% tricine gels). They were then transferred onto nitrocellulose (NC) membranes at 100 V for 1.5 h and blocked using 5% non-fat milk in Tris-buffered saline with 0.1% Tween-20 (TBST) for 1 h. Then, the NC membrane was incubated with the primary antibodies and second antibody, respectively. Target protein bands were visualized on the membrane using the ECL western blotting detection reagents. β-actin was used as an internal control of equal proteins loading and transfer. The proteins expression was quantified by Quantity-One Software, and the expression rate was labeled under the band [33].

### Animal Model

BALB/c male nude mice (~ 17 g) were purchased from the Model Animal Research Center of Nanjing University and bred in an axenic environment (specific pathogen-free animals). Tumor models were established by subcutaneous injection of cell suspension (SGC-7901 cells, 100 μL, 1 × 10^6^/mL) into the shoulder of the nude mice. Bright images of tumor were generated 15 days after the subcutaneous injection tumor cells. All animal experiments were conducted in accordance with the protocols approved by Anhui Agricultural University Laboratory Animal Center (Permit Number: SYXK 2016-007). A vernier caliper was used to determine the maximum longitudinal diameter (length) and maximum transverse diameter (width) of each tumor. The tumor volume was then calculated using the formula length × width^2^ × 0.5 [34].

### In Vivo Anticancer Study

Follow-up tests were performed when the tumor volume reached 50 mm^3^. Mice randomly divided into five groups were subjected to the following intravenous treatments: PBS (100 μL), Morin, Morin–liposomes, Au-Apt, and Apt-Au@MSL. The mice were injected intravenously into the tail at a dose of 2 mg/kg of the material for treatment. The tumor sizes of the mice were measured after 24 days. The body weight and survival of the mice were also determined. The mice were euthanized, and tumor and organ tissues of the mice were collected for H&E staining [35, 36].

After blocking and permeabilization, the tumor slides were washed with PBS, stained with TUNEL assay, and subjected to DAPI counterstaining. Fluorescence images were obtained by CLSM. For DAPI imaging, Ex = 358 nm and Em = 461 nm, and for TUNEL assay, Ex = 450–500 nm and Em = 515–565 nm [37].

### Toxicity Evaluate Assay

To evaluate the toxicity of Apt-Au@MSL treatment on vital organs, the mice in the four treatment groups were sacrificed after 30 days. The important organs (liver, spleen, kidneys, heart, lungs, and tumor) of the mice from all groups were collected and stained with H&E. Blood samples were also collected, and blood glucose were measured. In addition, the weights of the mice were determined [38].

## Results and Discussion

### Characterization

The present study aimed to develop a novel liposome that possesses the advantages of nanoparticles (Apt-loaded Au NPs) and can be effectively used as an anticancer agent. The basic properties of the new liposomes are important to conduct further studies. Apt-Au@MSL was synthesized as described in a previous study [23], with minor modifications, and then characterized using various methods (Fig. 3). Figure 3b presents the results of UV–vis spectroscopy, which shows the characteristic peak and can monitor the formation of Au NPs. Characteristic peaks were observed in the UV–vis spectrum of Au NPs at approximately 530 nm. Specifically, UV–vis spectra analysis exhibited strong UV absorbance, which was influenced by surface Apt modification. The same results were observed. Characteristic peaks were displaced after pH-sensitive liposomes were coated with Morin. The characteristic peak of the fabricated Apt-Au@MSL was located at 362 and 550 nm. These results indicated that synthesis was successfully completed. For further characterization, FT–IR spectroscopy was conducted to verify the results of the synthesis. The characteristic peaks red shift of Morin–liposomes and Apt-Au@MSL further indicated the successful modification of Morin (Fig. 3c). We performed Morin release assay in PBS at different pH levels to determine the pH sensitivity of Apt-Au@MSL. These three pH values simulate the neutral environment of blood circulation, the mildly acidic environment of tumor, and the acidic environment of the intracellular body. The release percentage of Morin was detected by UV–vis spectroscopy. At pH 5.0, about 54% of Morin was released within 24 h, and continuous release was observed in the following 96 h; meanwhile, at pH 7.4, only about 10% of Morin was released within 24 h. These results show that Apt-Au@MSL exhibits good stability under normal physiological conditions, preventing drug leakage; however, the drug is released quickly in the nuclear body. This drug release behavior can effectively improve the effect of treatment (Fig. 3d). TEM and SEM were conducted to elucidate the structures of the liposome and Apt-Au@MSL (Fig. 3f). TEM was conducted to elucidate the structures of the AuNPs. As shown in Fig. 3g, the AuNPs have a particle size of about 10 nm, which is consistent with the size of the modification on the liposomes (Fig. 3g). The SEM results in Fig. 3e indicate that raw liposomes solely form unilamellar vesicles with a nonuniform diameter of about 120 nm, which is consistent with the size determined by DLS. By contrast, Apt-Au@MSL shows that the morphology of hybrid vesicles is attributed to Au-Apt modification. The diameter of 150 nm was also consistent with the size determined by DLS (Fig. 3i). Notably, TEM investigation of the interactions between the pH-sensitive liposomes and Au-Apt revealed that the Au NPs were associated almost exclusively with the vesicular lipid bilayer and localized at the periphery of the vesicles (Fig. 3h). The ζ-potential measurements are presented in Fig. 3j, and the zeta potentials of Au NPs and Au-Apt are negative. The result shows that both Au NPs (− 57.1 ± 0.3 mV) and Au-Apt are negatively charged (− 31.7 ± 0.2 mV). Apt-Au@MSL was modified with positively charged Morin–liposomes, and the potential changed to 36.4 ± 0.3 mV. Positive and negative charge adsorption is the mechanism by which Au-Apt and Morin–liposomes bind. In addition, Fig. 3k represents the change in the encapsulation rate of the particle over time, indicating the stability of the particle over a period of time. As shown in Fig. 3k, the encapsulation rate of the particle hardly changes within 24 h, indicating that the particle exhibits good stability over a period of time. The standard curve of Morin concentration versus the UV-Vis absorbance of Morin was prepared to study the encapsulation rate of Morin–liposome and Apt-Au@MSL. The results revealed that the entrapment efficiency of Morin–liposome and Apt-Au@MSL can reach 90.2% and 89.6%, as shown in Table S1 and Fig. S1

### Anticancer Activity Test of Apt-Au@MSL

#### In Vitro

**Fig. 4:**
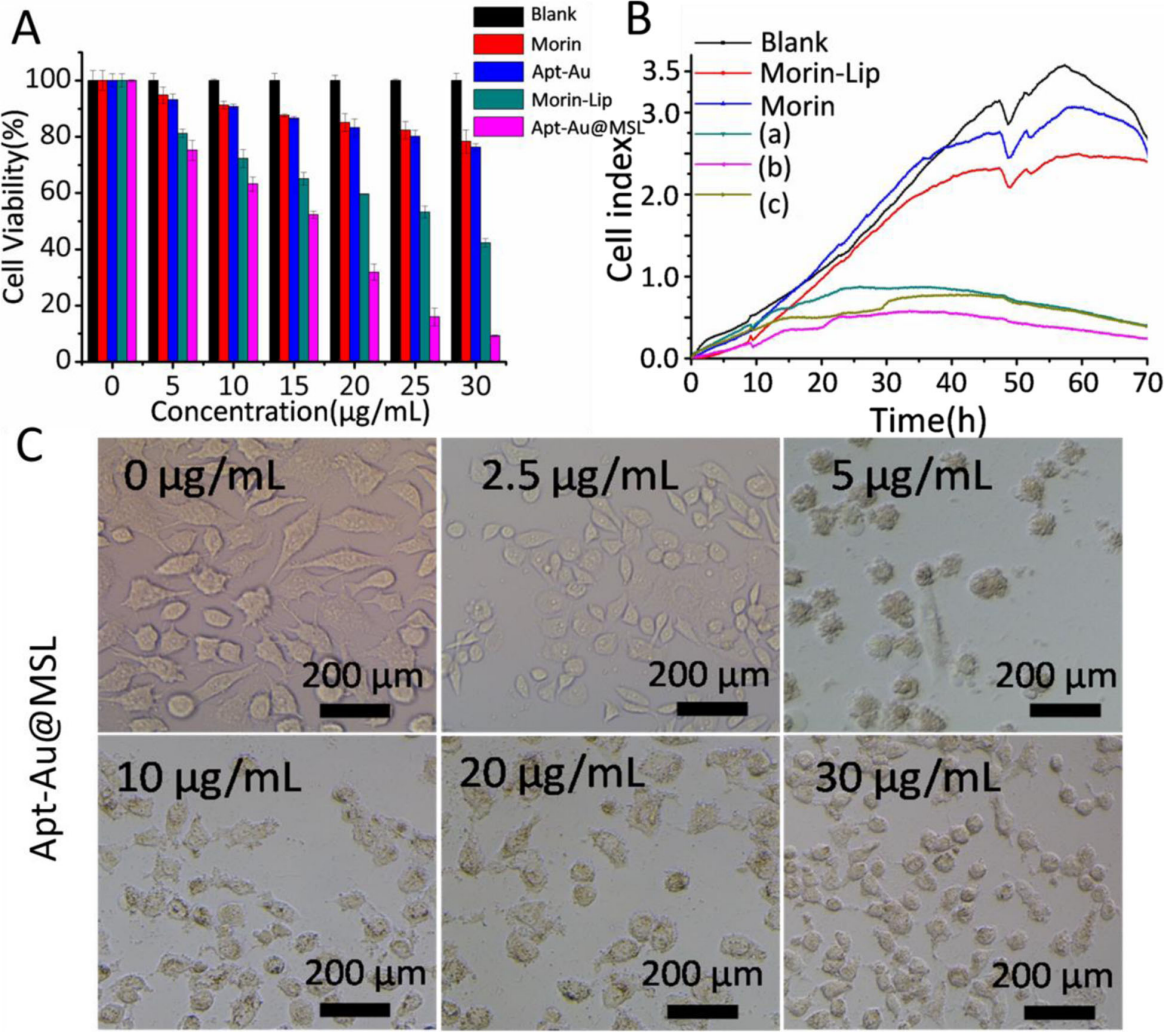
**a** Cell viability of SGC-7901 cells incubated with different material (Morin, Au-Apt, Morin–liposome, and Apt-Au@MSL) at different concentrations (0, 5, 10, 15, 20, and 30 μg/mL). The cell has treated by PBS was set as blank group. **b** The cell proliferation curve of SGC-7901 cells was detected by RT-CES system. The different compounds were added at 10 h. The concentrations of **a**, **b**, and **c** are different Apt-Au@MSL (10, 20, and 30 μg/mL), respectively. **c** The bright cell images at the different concentrations (0, 2.5, 5, 10, 20, and 30 μg/mL)

Morin exhibits superior antitumor activity but has low bioavailability because of its low water solubility. After Morin was encapsulated by pH-sensitive liposomes and transformed into a water-soluble material, the surface of the liposome was modified using Au-Apt to obtain the targeted antitumor effect. Figure 4a shows the anticancer activity of Apt-Au@MSL by MTT assay in vitro. Drug concentration is defined by Morin concentration. The Morin-treated group showed no apparent anticancer activity, compared with the blank group. However, the group treated with Morin encapsulated by pH-sensitive liposomes exhibited enhanced anticancer activity. The result suggests that the antitumor activity of Morin–liposomes was superior to that of raw Morin. Simultaneously, we found that the antitumor activity of the liposome modified by Apt (Apt-Au@MSL) was improved. Table 1 lists the IC_50_ values of Morin, Morin–liposomes, Apt-Au@MSL, and cisplatin. Morin, Morin–liposomes, and Apt-Au@MSL exhibited a broad range of anticancer activity on tumor cells. They showed a distinct preference for SGC-7901 cells with high potency and low toxicity toward normal human cells. The IC_50_ value of Apt-Au@MSL was 15.6 ± 1.5 μg/mL for the SGC-7901 cells. These results were further confirmed by RTCA testing. The SGC-7901 cells were cultured with Apt-Au@MSL at different concentrations; the blank group was treated with PBS, and the control group consisted of Morin and Morin–liposomes. Adhesion and spreading were monitored using iCELLigence (Fig. 4b). The results were largely similar to those obtained by MTT testing. The antitumor activity of Apt-Au@MSL was significantly higher than those of Morin and Morin–liposomes. Apt-Au@MSL could inhibit the proliferation of SGC-7901 cells with an increase in concentration. Changes in cell morphology after Apt-Au@MSL treatment were also observed by fluorescence microscopy. As shown in Fig. 4c, the SGC-7901 cells without Apt-Au@MSL maintain the structural integrity of the cell. By contrast, cell integrity is compromised after cells are treated with Apt-Au@MSL at different concentrations.

**Table 1:** IC_50_ values of Morin, Morin–liposomes, and Apt-Au@MSL in various human cancer cells

Complex	IC_50_ (μg/mL)				
	HeLa	BGC-823	SGC-7901	A549	Hs68
Morin	100	89.7 ± 3.9	83.8 ± 2.6	79.3 ± 3.8	> 100
Morin-lip	68.2 ± 3.2	58.7 ± 5.1	47.1 ± 5.1	63.2 ± 1.9	> 100
Apt-Au@MSL	36.8 ± 3.6	35.4 ± 1.7	15.6 ± 1.5	45.6 ± 2.8	> 100
Cisplatin	9.5 ± 0.3	10.6 ± 1.2	5.3 ± 0.9	16.3 ± 0.9	3.8 ± 3.7

### Apoptotic Cell Death Analysis

**Fig. 5:**
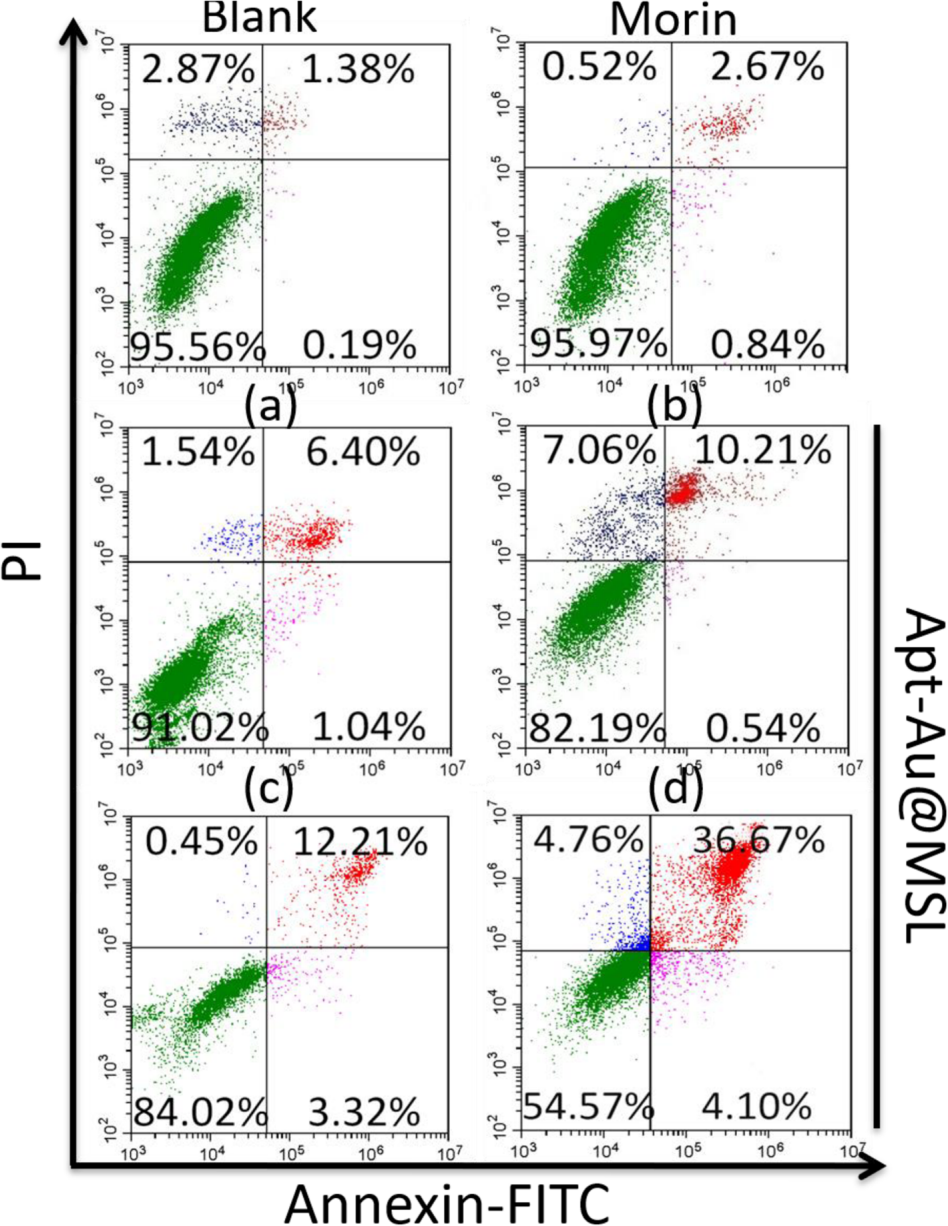
Annexin V-FITC/PI staining-based flow cytometry analysis of SGC-7901 apoptosis after treating with different methods. The concentration of **a**, **b**, **c**, and **d** is 5, 10, 20, and 30 μg/mL, respectively

Quantitative analysis of apoptosis was performed by flow cytometry using Annexin–FITC staining. The cells were stained with PI and Annexin V–FITC and then analyzed using a CytoFLEX flow cytometer (Beckman Coulter). Annexin V was used to detect early apoptotic cells bound to the exposed phosphatidylserine, and PI labeling was used to stain the late apoptotic cells. The apoptosis ratio was 1.57% for the blank groups. The cells treated with Morin showed an apoptosis ratio of 3.51%. Apt-Au@MSL at different concentrations exhibited a higher inducing capability with apoptosis ratios of 7.44%, 10.75%, 15.53%, and 40.77% (Fig. 5). The enhanced apoptosis also confirmed the outstanding anticancer activity induced by Apt-Au@MSL. The apoptosis ratio of the SGC-7901 cells increased with increased Apt-Au@MSL concentration.

### Anticancer Activity Study by Fluorescence Assay

**Fig. 6:**
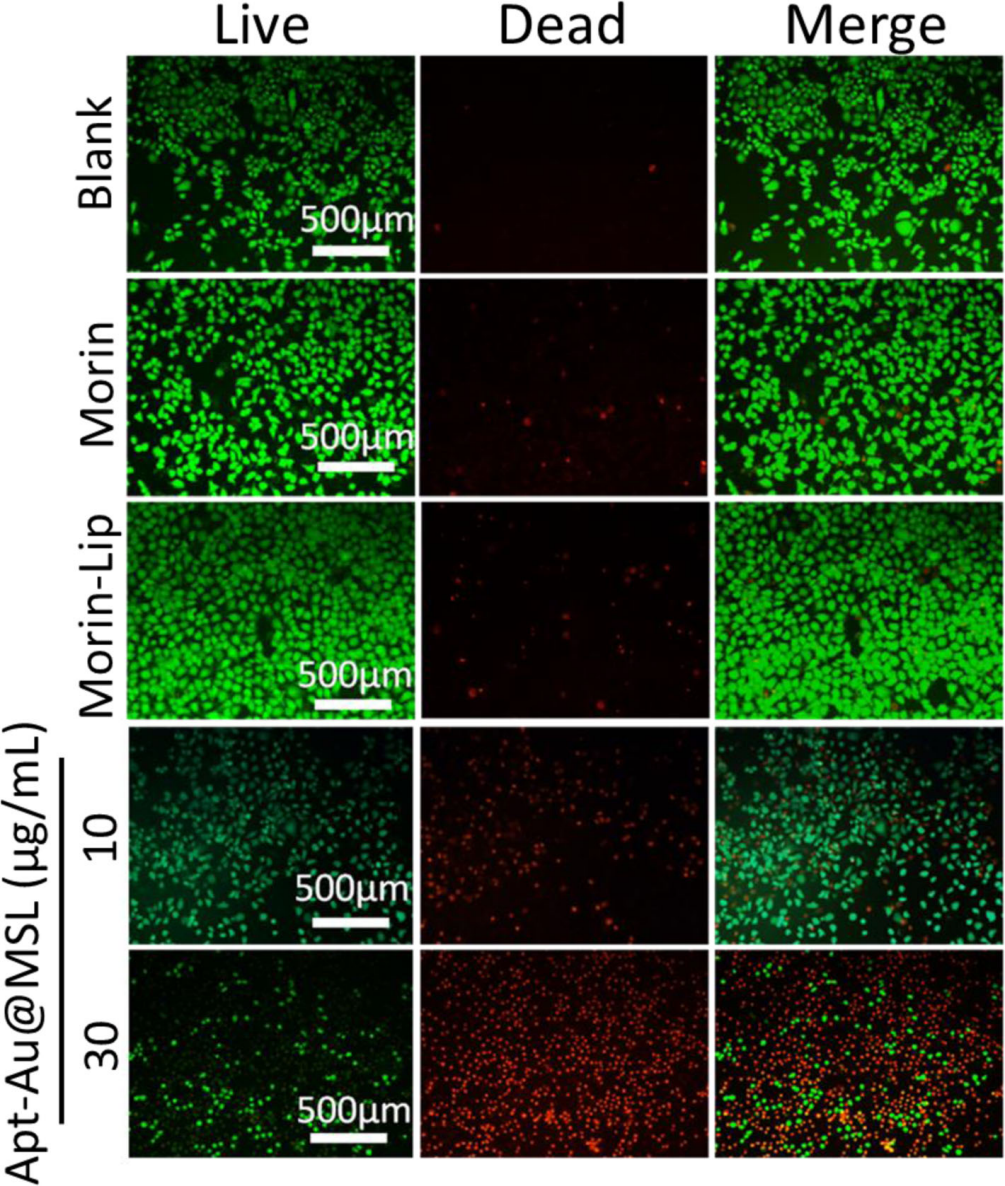
Fluorescence microscopic images of SGC-7901 incubated with different concentration Apt-Au@MSL and subsequent brief staining. The blank group was PBS. The Morin and Morin–liposome were set as control group

The antitumor effect on SGC-7901 cells induced by Apt-Au@MSL was intuitively evaluated by LIVE/DEAD fluorescence assays. After cells were incubated with Morin, Morin–liposomes, and Apt-Au@MSL at different concentrations, they were co-stained with the LIVE/DEAD kit for 30 min under dark conditions. In Fig. 6, the cells in the blank group show that all of the green fluorescence cells represent live cells. The control groups, including Morin and Morin–liposomes, show the number of red fluorescent cells. However, the cells that were treated with Apt-Au@MSL at different concentration clearly exhibited a large number of apoptotic cells. With an increase in concentration, live cells gradually decreased. The percentage of death cells increased predominantly, and cell density decreased. The result confirmed that Apt-Au@MSL can effectively promote tumor apoptosis.

### Cell Integrity Study

**Fig. 7:**
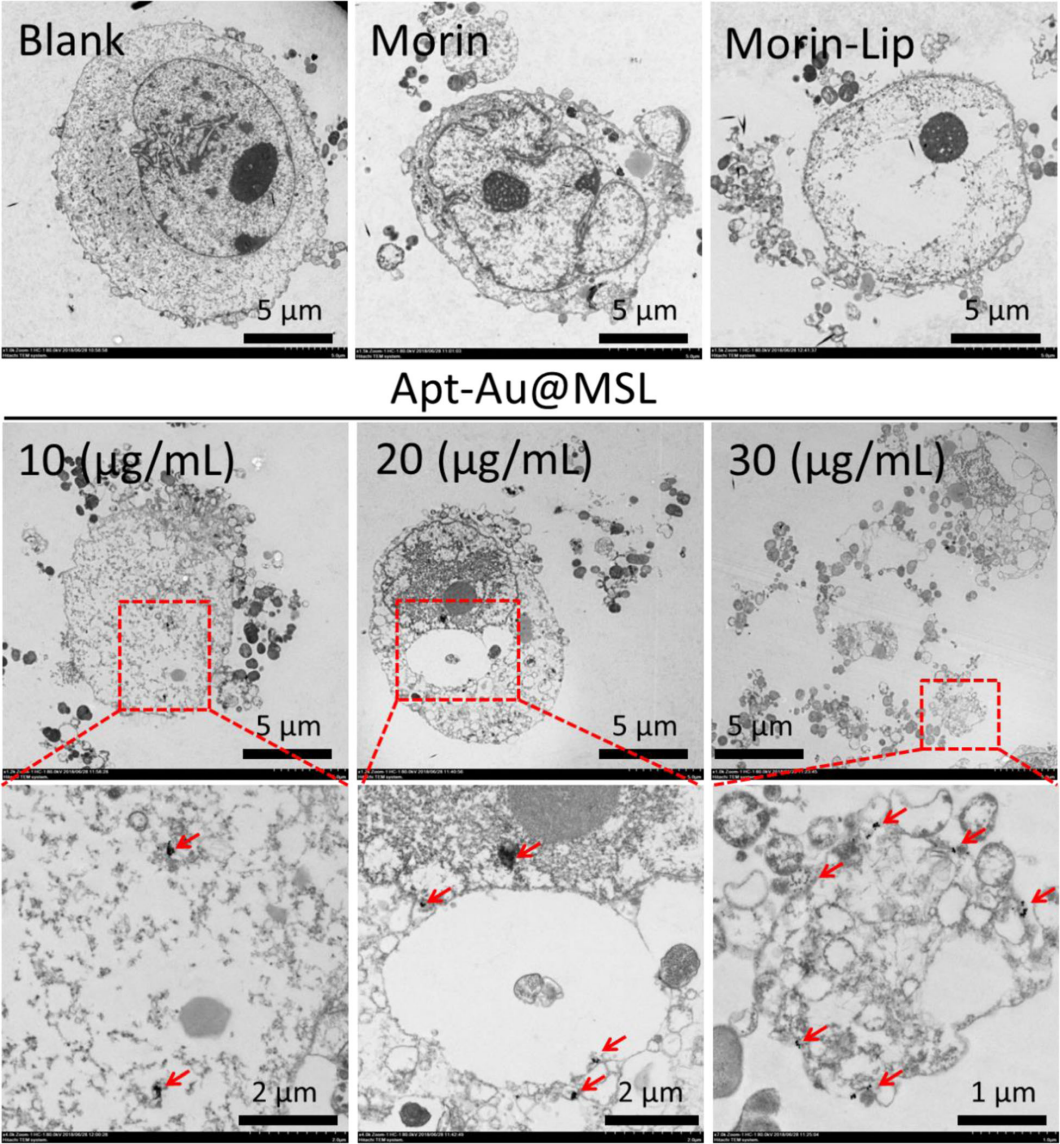
TEM images of SGC-7901 cells treated with different concentration of Apt-Au-MSL (10, 20, and 30 μg/mL). The blank group was PBS. The Morin and Morin–liposome were set as control group. The red square is the enlarge area. The red arrow point to Au NPs

To confirm the influence of uptake and transport of the Apt-Au@MSL to the SGC-7901 cell, we conducted TEM assay. The change in cell morphology was observed by TEM. TEM images were conducted to observe the internal structure of the cells and provide a reference for anticancer mechanisms. As shown in Fig. 7, the blank images of the SGC-7901 cells without treatment exhibited significant changes in the morphology and appearance of clear cell walls. In the control group (Morin and Morin–liposomes), the cell morphology showed partial damage, and the nuclear region contracted. However, significant changes in the cell wall and internal structure were also observed after exposure to Apt-Au@MSL. The cytoplasm leaked, and the nuclear structure became unclear. Many cell fragments were formed around hollow cells. In addition, the cell walls disintegrated or were destroyed. Entire profiles became unclear, cells were damaged, and the cytoplasm leaked. The red arrows represented the Au NP area. As the concentration of the Apt-Au@MSL increased, more Au NP regions appeared in the nucleus. This occurrence suggested the release of Morin after Apt-Au@MSL entered the cell interior, hence the appearance of a large amount of Au NPs. These aforementioned results suggest that antitumor activity was associated with compromised cell integrity and nuclear structure.

### Molecular Mechanism Induced by Apt-Au@MSL

**Fig. 8:**
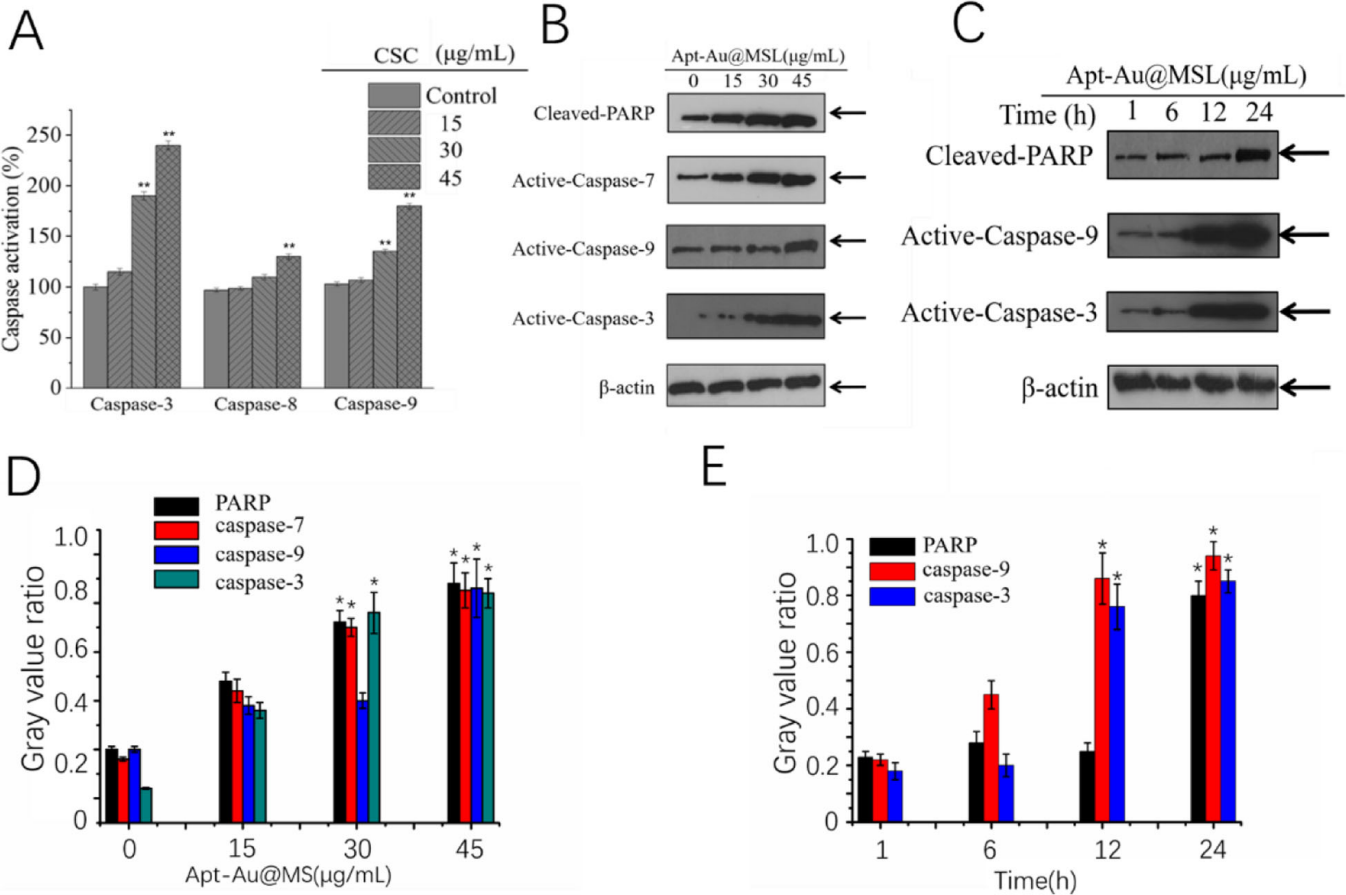
Apt-Au@MSL induced apoptosis in SGC-7901 cells. **a** SGC-7901 cells were treated with indicated concentrations of Apt-Au@MSL for 24 h. Then, the total protein was extracted and incubated with synthetic Apt-Au@MSL substrates for measuring caspase activities. Dose-dependent (**b**) and time-dependent (**c**) effects of Apt-Au@MSL on PARP and caspases expression. **d**, **e** Quantitative analysis of PARP, caspase-7, caspase-9, and caspase-3 expressions. Data are means ± SD, *P < 0.05, **P < 0.01

To explore the molecular mechanism of Apt-Au@MSL-induced apoptosis, we detected the expression levels of caspases and PARP. Activation of caspase-3, -8, and -9 was first detected with specific substrates. As shown in Fig. 8a, Apt-Au@MSL treatment induces dose-dependent activation of caspase-3, -8, and -9. Caspase-9 promotes mitochondria-mediated apoptosis more than does caspase-8, indicating that the mitochondria-mediated apoptosis signaling pathway is dominant. Western blot analysis further confirmed the existence of Apt-Au@MSL-induced apoptosis at the protein level. Figures 8b and c show that after cells are treated with Apt-Au@MSL, activation of caspases and cleavage of PARP shows time and dose dependence (Figs. 8b, c). In Fig. 8d, e, the Western blot analysis confirmed our results. In conclusion, Apt-Au@MSL inhibits the growth of SGC-7901 cells mainly by inducing apoptosis.

### Apt-Au@MSL Inhibits Tumor Growth

#### In Vivo

**Fig. 9:**
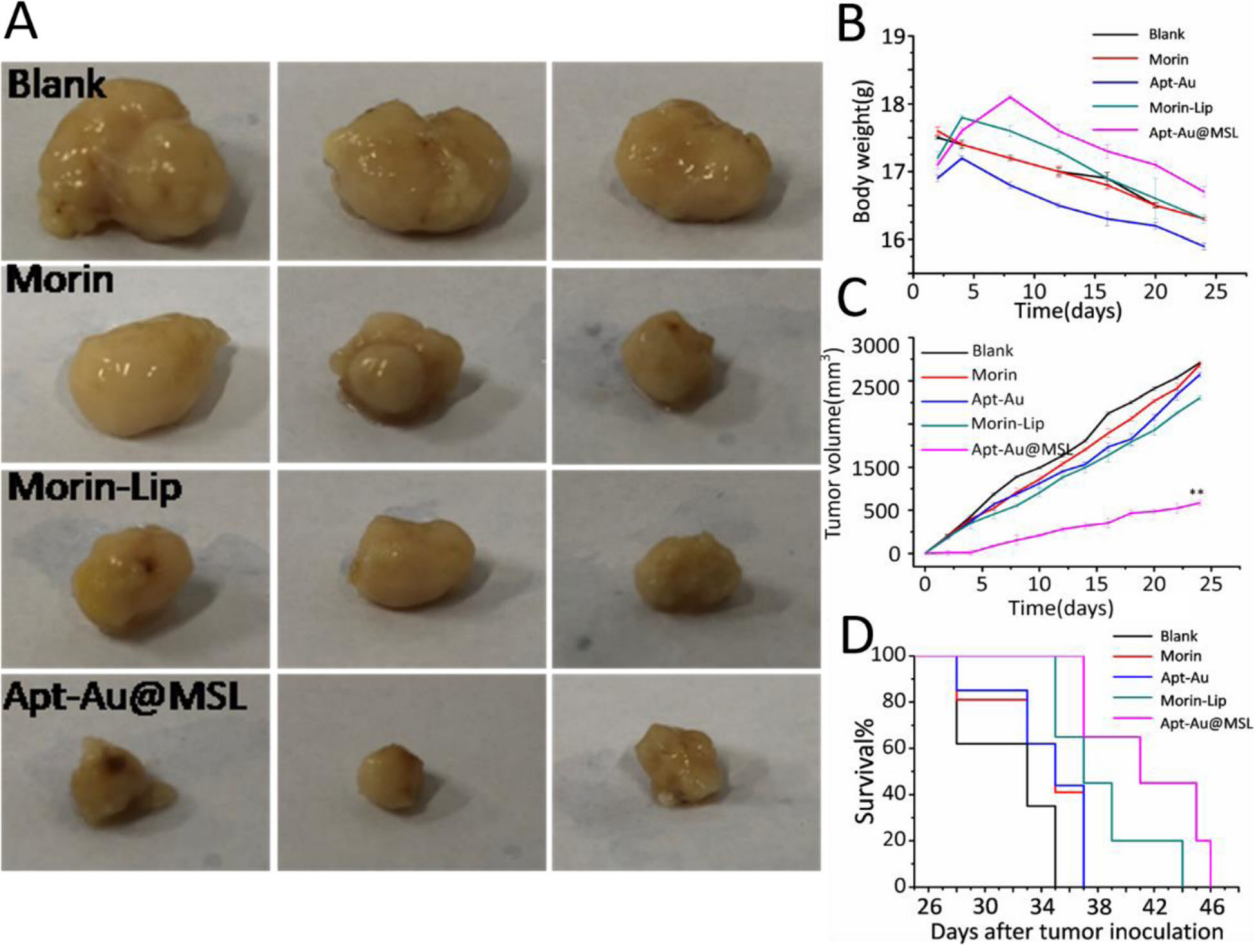
**a** In vivo applications of Apt-Au@MSL and photographs of the mice tumor taken 24 days. A dosage of 2 mg/kg was administrated intravenously for all mice (*n* = 6–8). **b** Tumor weight of mice in different groups after 24 days. **c** Tumor volume index for the different treatment groups. The tumor sizes were measured at the indicated time points. **d** Survival rate of the mice in different group after tumor inoculation. Data are means ± SD (*n* = 6-8). The intravenous injection of PBS was set as blank group (100 μL); the treated by Morin and Morin–liposome were set as control group. In vivo therapeutic effects of Apt-Au@MSL in SGC-7901-bearing mice. Data are means ± SD, *P < 0.05, **P < 0.01

The in vivo antitumor activities of Apt-Au@MSL were evaluated using an SGC-7901 tumor xenograft model. A comparison of the images of the tumors with those of the control group (Morin and Morin–liposomes) showed that mice treated with Apt-Au@MSL markedly reduced the weight and size of the tumor (Fig. 9a). The relative tumor volume curves and the mice weight curves indicate that the Apt-Au@MSL in vivo exhibits a higher anticancer efficiency (Fig. 9b) than those of the other treatment groups. No significant difference in average tumor volume was indicated between the control group (Morin and Morin–liposomes) and the blank group. The tumor volume of the Apt-Au@MSL group was only nearly a tenth of the blank group and nearly a sixth of the control group. The result indicated that raw Morin and Morin–liposomes at 40 mg/kg exerted no effect on the growth rate of tumors. However, Apt-Au@MSL could inhibit tumor growth. The body weight of mice in the different groups (PBS, Morin, Morin–liposomes, and Au-Apt groups) showed no marked fluctuation (Fig. 9c) during the treatment period. This result suggested that the treatment was tolerated and caused no acute side effects. Notably, we found that the mice with Apt-Au@MSL treatment showed markedly prolonged survival (Fig. 9d). The surviving mice in this group behaved normally, showing no apparent sign of unhealthy condition. These results demonstrated that the administration of Apt-Au@MSL could inhibit tumor growth in xenograft mouse models.

### Histological Analysis of Anticancer Activity

**Fig. 10:**
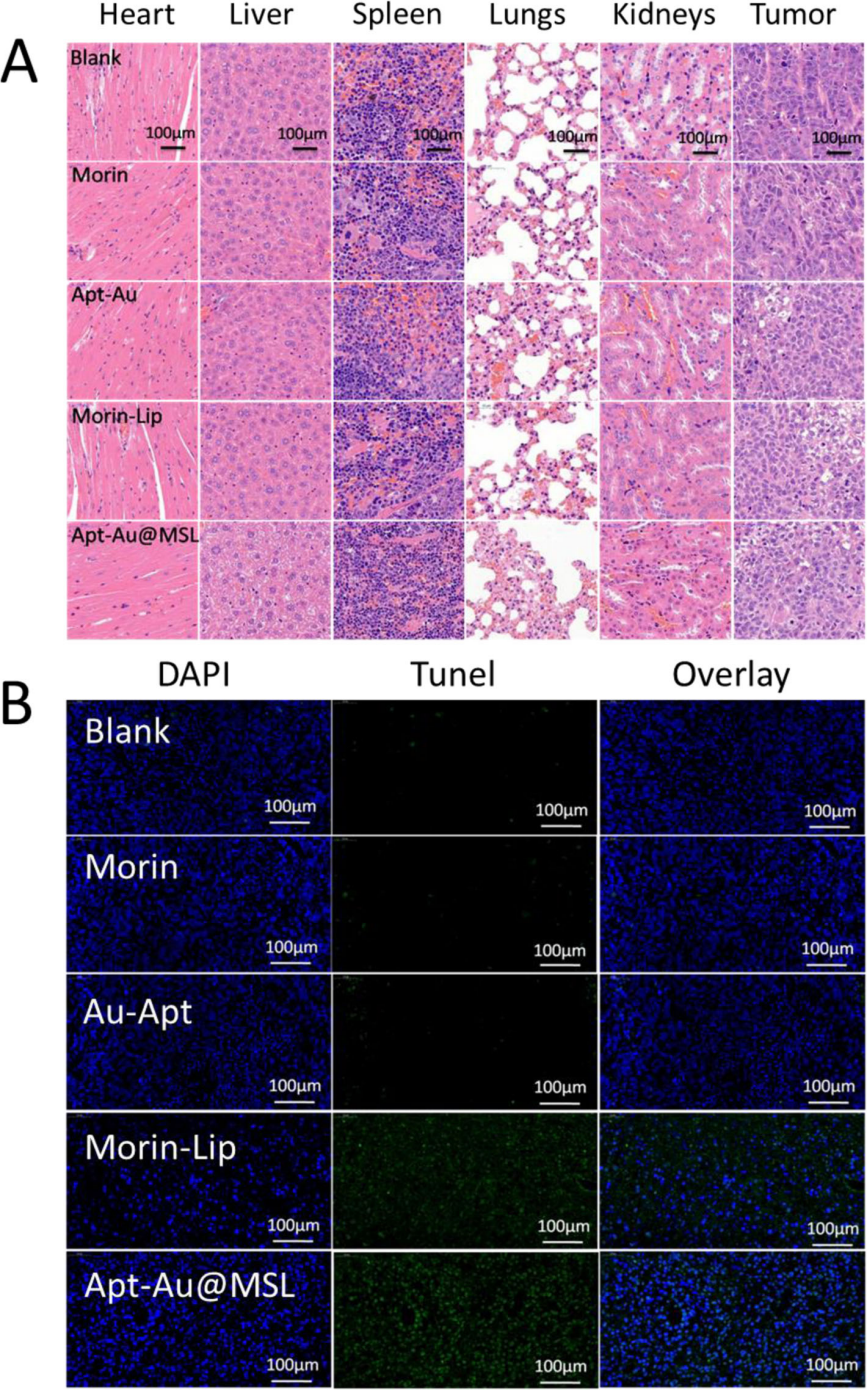
**a** H&E staining analysis of the tumors in mice. Histological analysis of the tumors in mice following different treatments as PBS, Morin, Morin–liposome, Au-Apt, and Apt-Au@MSL group. **b** Apoptotic cells were detected by a TUNEL assay (green) and co-stained by nuclear staining DAPI (blue)

H&E staining of tumor tissue and organ samples was conducted after fixation and treatment. Treatment efficacy with respect to tumor cell death was also evaluated by H&E staining of tumor tissue from different groups. In Fig. 10a, the mice treated with Au-Apt, Morin, and Morin–liposomes show the same extent of thermal damage as that of the mice in the blank group. No apparent apoptosis was observed in the blank group. The tumor tissue sections consisted of tightly packed tumor cells. However, Apt-Au@MSL treatment exhibited the most significant damage to the tumor tissue, with moderate cell apoptosis in the tumor. The result suggested anticancer activity in the mouse models treated with Apt-Au@MSL. To further investigate the ratio of apoptotic cells in tumors tissue in vivo, TUNEL assay was performed for the detection of apoptotic cells. As shown in Fig. 10b, the apoptotic cells in tumors can be stained with green fluorescence to indicate apoptosis. The merged images show fewer green fluorescent regions in the blank group and the control group (Morin and Au-Apt), indicating the presence of fewer apoptotic cells. The cells of the mice treated with Morin–liposomes appear partly apoptotic. Moreover, a large number of green fluorescent regions were observed in the group treated with Apt-Au@MSL, indicating a large amount of apoptotic cells. The results were consistent with that of H&E staining, confirming that Apt-Au@MSL can promote tumor apoptosis in vivo*.*

### In Vivo Toxicity Evaluation

**Fig. 11:**
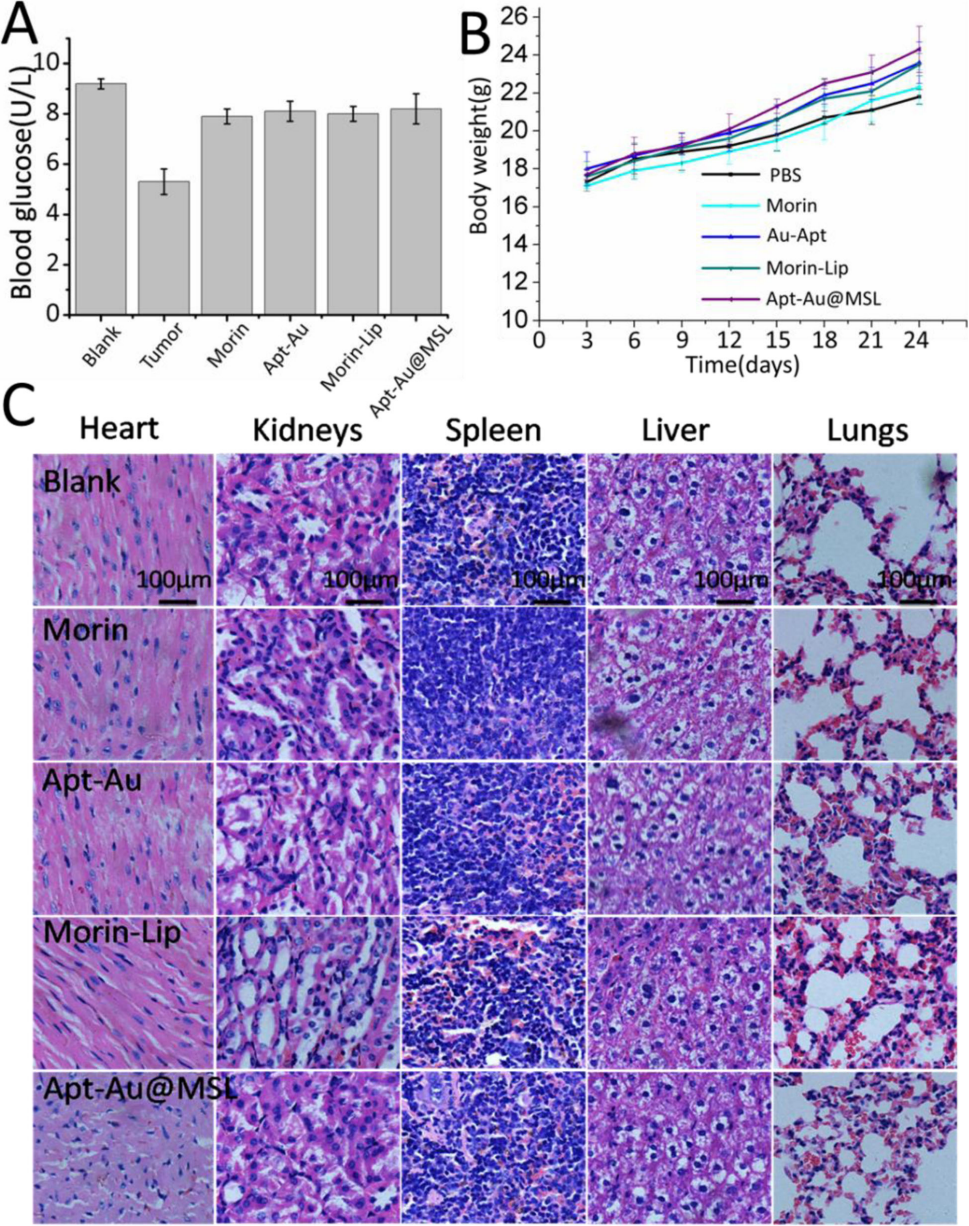
In vivo toxicity evaluation. **a** Blood glucose data detected in the mouse toxicity model. **b** Weight of mice in different groups after 24 days. **c** Images of H&E-stained major organs. Each value represents the mean ± SD (*n* = 3)

The potential in vivo toxicity is often a significant concern for the clinical application of anticancer medicine. To verify the applicability of Apt-Au@MSL in vivo, the mice were evaluated under different treatments (Morin, Morin–liposomes, Au-Apt, and Apt-Au@MSL). Blood biochemical assays were also conducted to examine possible changes in the biochemistry of mice after treatment. As shown in Fig. 11a, the blood glucose index for blood function of the Apt-Au@MSL groups was similar to those of the blank and control groups. No difference in body weight was found in each group. A steady increase was observed, indicating that the drug exhibited no toxicity (Fig. 11b). H&E staining of organ sections (Fig. 11c) showed no sign of damage or inflammation in the group treated with Apt-Au@MSL, compared with the blank and control group. This finding indicated that PBS, Morin, Morin–liposomes, Au-Apt, and Apt-Au@MSL were negligible side effects in vivo. These results, as well those of H&E staining, further indicate safety in the use of Apt-Au@MSL for tumor treatment.

## Conclusions

In conclusion, this study presents the synthesis of an antitumor nanomaterial, Apt-Au@MSL. Apt-Au@MSL exhibited excellent monodispersity and tumor-targeting properties. The polarity of Morin was modified, and the antitumor activity was enhanced. The pH of the solution was 5.0, and the release rate of Morin from Apt-Au@MSL was the maximum in the characterization experiments. Apt-Au@MSL showed that the morphology of hybrid vesicles was attributable to Au-Apt modification. The diameter of 150 nm was consistent with the size determined by DLS. We screened our model cancer cell by MTT assay and found that SGC-7901 cells could effectively suppress proliferation. The IC_50_ of Apt-Au@MSL was 15.6 ± 1.5 μg/mL for the SGC-7901 cells. Fluorescent flow cytometric assays confirmed that Apt-Au@MSL could be used as an effective anticancer material and induced apoptosis in vitro. The Apt-Au@MSL found in the internal cell, as shown in the TEM images, suggested that Apt-Au@MSL could target the cancer cell. The administration of Apt-Au@MSL could inhibit tumor growth in xenograft mouse models, as determined from tumor weight testing. H&E staining and TUNEL assay further confirmed that Apt-Au@MSL could promote tumor apoptosis in vivo*.* Both blood biochemistry testing and H&E staining suggested that these materials exhibit negligible acute toxicity and good biocompatibility in vivo.

## Supplementary information

**Additional file 1.** Supplementary figures and tables.

## Data Availability

The authors declare that the materials, data, and associated protocols are promptly available to the readers without undue qualifications in material transfer agreements. All data generated and analyzed during this study are included in this article.

## Abbreviations

Apt: Aptamers
Apt-Au: Aptamers and Au nanoparticle
MSL: Morin pH-sensitive liposome
AuNPs: Gold nanoparticles
ROS: Reactive oxygen species
Apt-Au@MSL: Aptamers and Au nanoparticle-modified Morin pH-sensitive liposome
PC: L-α-Phosphatidylcholine
Chol: Cholesterol
CHEMS: Cholesteryl hemisuccinate
PVP: Polyvinylpyrrolidone
PI: Propidium iodide
FT–IR: Fourier-transform infrared spectroscopy
UV–Vis: Ultraviolet–visible spectroscopy
TEM: Transmission electron microscopy
SEM: Scanning electron microscopy
DLS: Dynamic light scattering
PDI: Polydispersity index
ATCC: American Type Culture Collection
CLSM: Confocal laser scanning microscopy
TUNEL: TdT-mediated dUTP Nick-End Labeling
H&E staining: Hematoxylin-eosin staining
RTCA: Real-time unlabeled cell analysis
