# Supplementary material for: Designing Aptamer-Gold Nanoparticle-Loaded pH-Sensitive Liposomes Encapsulate Morin for Treating Cancer
Source: Nanoscale Res Lett. 2020 Mar 30;15:68. doi: 10.1186/s11671-020-03297-x (PMC7105578; doi:10.1186/s11671-020-03297-x)
Supplement: Supplementary file 1 — Additional file 1. Supplementary figures and tables. [file 11671_2020_3297_MOESM1_ESM.docx]

Designing Aptamer-Gold Nanoparticle Loaded pH-Sensitive Liposomes Encapsulate Morin for Treat Cancer

**Xiaoyuan Ding^1*^, Chenyang Yin^1*^, Weiwei Zhang^2^, Yu Sun^1^, Zhenzhen Zhang^1^, Endong Yang^1^, Dongdong Sun^1*^, Weiyun Wang^1*^**

^1^ School of Life Sciences, Anhui Agricultural University, Hefei, 230036, China

^2^ School of Biochemical Engineering, Anhui Polytechnic University, 8 Zheshan Road, Wuhu, Anhui 241000, China

^*^Corresponding author. Tel.:086 551 65786703; fax: 086 551 65786703

E-mail addresses: Dongdong Sun: sunddwj@126.com , Weiyun Wang: weiywswzy@163.com

^*^Both authors contribute equally to this work.

Entrapment efficiency rate (EE%) was determined using UV-Vis spectra with the principle of penetration. The standard curve of morin was made by respectively testing the absorbance of various concentrations (80, 70, 60, 50, 40, 30 μg/mL) and mapping analysis. Briefly, morin-liposomes, Apt-Au@MSL(100μg/mL,1.5mL) was eluted with PBS (pH 7.4) through a centrifugal (7000 rpm, 5min) to separate free drug and the supernatant was detected with scanning wavelength. The entrapped drug was determined by disrupting the vesicles with Triton X-100 and measuring absorbance at 385 nm by UV-spectrophotometer (UV–Vis, S-3100 Photodiode Array, Scinco Co., Ltd., Korea). Entrapment efficiency was determined by the ratio of the actual amount of encapsulated drug over the total amount of drug used for the preparation of liposomes.

Table S1: The encapsulation rate of morin-liposome and Apt–Au@MSL.

| formulation code | Absorbance | Concentration (μg/mL) | EE (%) |
| --- | --- | --- | --- |
| Morin-Lip | 0.278 | 24.52 | 90.2% |
| Apt-Au@MSL | 0.295 | 25.93 | 89.6% |

Data are average values of at least three replicates.


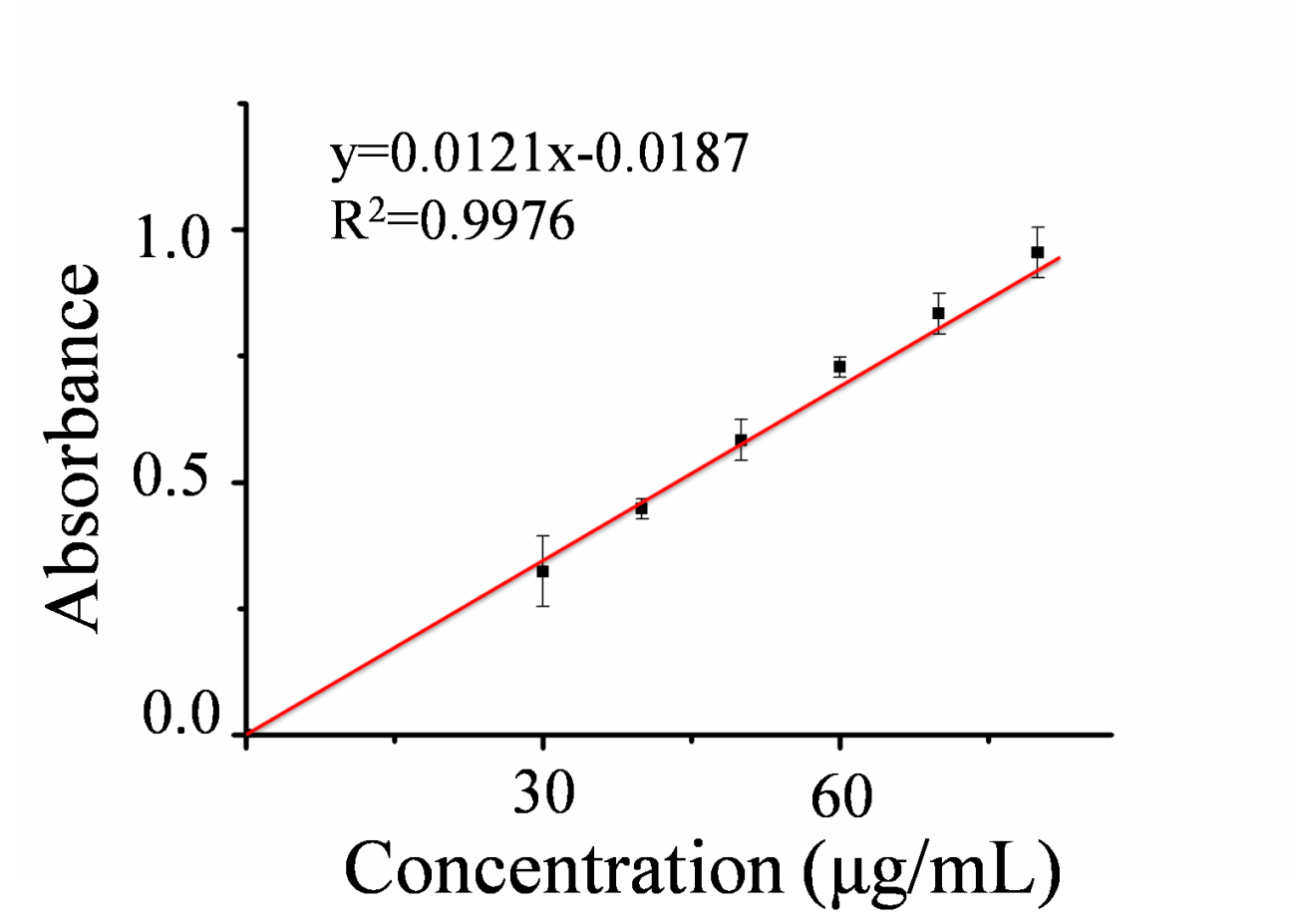


Fig. S1. Standard curve of Morin through UV-Vis absorbance.


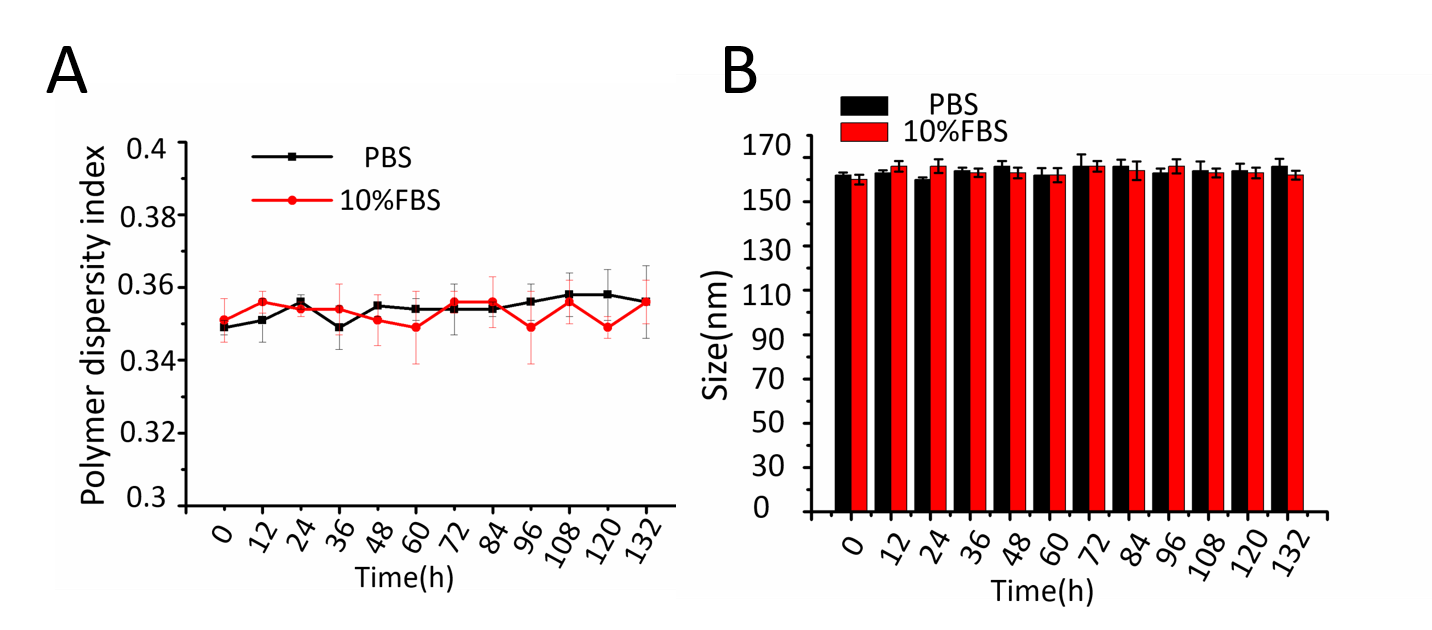


Fig. S2. The stability analysis of the Apt-Au@MSL at different biological conditions. (A) Changes in the Polymer dispersity index of Apt-Au@MSL in PBS (100 µM) and 10% FBS. (B) Changes in the hydrodynamic size of Apt-Au@MSL in PBS (100 µM), and 10% FBS.
